# Discovering lethal alleles across the turkey genome using a transmission ratio distortion approach

**DOI:** 10.1111/age.13003

**Published:** 2020-10-01

**Authors:** E. A. Abdalla, S. Id‐Lahoucine, A. Cánovas, J. Casellas, F. S. Schenkel, B. J. Wood, C. F. Baes

**Affiliations:** ^1^ Centre for Genetic Improvement of Livestock, Department of Animal Biosciences University of Guelph Guelph ON N1G 2W1 Canada; ^2^ Departament de Ciència Animal i dels Aliments Universitat Autònoma de Barcelona Bellaterra 08193 Spain; ^3^ Hybrid Turkeys C‐650 Riverbend Drive, Suite C Kitchener ON N2K 3S2 Canada; ^4^ School of Veterinary Science University of Queensland Gatton Qld 4343 Australia; ^5^ Institute of Genetics, Vetsuisse Faculty University of Bern Bern 3001 Switzerland

**Keywords:** fertility, functional analysis, gene set enrichment, lethal haplotypes, transmission ratio distortion

## Abstract

Deviation from Mendelian inheritance expectations (transmission ratio distortion, TRD) has been observed in several species, including the mouse and humans. In this study, TRD was characterized in the turkey genome using both allelic (specific‐ and unspecific‐parent TRD) and genotypic (additive‐ and dominance‐TRD) parameterizations within a Bayesian framework. In this study, we evaluated TRD for 23 243 genotyped Turkeys across 56 393 autosomal SNPs. The analyses included 500 sires, 2013 dams and 11 047 offspring (trios). Three different haplotype sliding windows of 4, 10 and 20 SNPs were used across the autosomal chromosomes. Based on the genotypic parameterizations, 14 haplotypes showed additive and dominance TRD effects highlighting regions with a recessive TRD pattern. In contrast, the allelic model uncovered 12 haplotype alleles with the allelic TRD pattern which showed an underrepresentation of heterozygous offspring in addition to the absence of homozygous animals. For regions with the allelic pattern, only one particular region showed a parent‐specific TRD where the penetrance was high via the dam, but low via the sire. The gene set analysis uncovered several gene ontology functional terms, Reactome pathways and several Medical Subject Headings that showed significant enrichment of genes associated with TRD. Many of these gene ontology functional terms (e.g. *mitotic spindle assembly checkpoint*, *DRM complex* and *Aneuploidy*), Reactome pathways (e.g. *Mismatch repair*) and Medical Subject Headings (e.g. *Adenosine monophosphate*) are known to be related to fertility, embryo development and lethality. The results of this study revealed potential novel candidate lethal haplotypes, functional terms and pathways that may enhance breeding programs in Turkeys through reducing mortality and improving reproduction rate.

## Introduction

Owing to its considerable economic impact, reproduction has drawn the attention of turkey breeders and producers (Saif & Nestor 2002; Huff *et al*. 2005; Emamgholi Begli *et al*. 2019). Lethal alleles may cause mortality before, during or after the embryonic stage, and hence reduce reproductive performance. By their nature, livestock breeding programs tend to increase inbreeding levels among individuals, and consequently the probability of mating parents carrying lethal alleles may increase (Granleese *et al*. 2015). Turkeys are not an exception, thus identifying genetic regions that influence reproductive efficiency and mortality is relevant and may enhance breeding programs in this species.

Many autosomal recessive lethal loci have been distinguished in livestock species such as cattle (e.g. Dong *et al*. 2019; Guarini *et al*. 2019) and the correct mate allocation is expected to reduce the economic losses (Cole *et al*. 2016). Several methods, such as screening for the absence of homozygous haplotypes (e.g. VanRaden *et al*. 2011; Hoff *et al*. 2017; Jenko *et al*. 2018) and transmission ratio distortion (TRD; Casellas *et al*. 2012, 2014), can be used to discover genomic regions with potentially lethal alleles. TRD is a process whereby the transmission of alleles from heterozygous parents to offspring deviates from Mendelian ratios, regardless of the cause (Crow 1999; Pardo‐Manuel De Villena *et al*. 2000; Huang *et al*. 2013). Thus, TRD reveals locus‐specific signals that provide insight into genetics and evolutionary processes of individual fitness variation, population divergence and speciation (Fishman & McIntosh 2019).

The availability of genomic markers has facilitated the task of investigating lethal alleles. The decline in reproductive performance and ability of parents to contribute equally to next generations may alter the expected Mendelian inheritance patterns, resulting in an observable TRD. For instance, it is possible to trace back the inheritance of each allele as well as the combination of two alleles inherited from parents to offspring using genotype trios with high accuracy. Several Bayesian models have been developed to detect and analyze all types of TRD based on allelic and genotypic parameterizations (Casellas *et al*. 2012, 2014). These two parametrizations have been already evaluated in a previous study (Casellas *et al*. In press) reporting the relevance of implementing and comparing the different parametrizations to capture all types of TRD. The objective of this study was to assess TRD based on allelic and genotypic parameterizations and perform a functional gene set enrichment analysis to uncover biological pathways associated with TRD in a purebred line of turkeys.

## Materials and methods

### Data

In this study, we used a turkey population with 23 243 (6867 males and 16 376 females) genotyped animals for 61 705 SNPs. The animals were hatched between late 2010 and early 2018, and genomic and pedigree information was provided by Hybrid Turkeys, Kitchener, Canada. The whole dataset combines 11 047 parent‐offspring genotyped trios, including 500 sires and 2013 dams. All birds were genotyped with the same SNP genotyping platform array (65 000 SNP; Illumina, Inc.) and mapped to the Turkey 5.0 *Meleagris gallopavo* assembly (Dalloul *et al*. 2010). Quality control analyses were performed and resulted in the removal of non‐autosomal SNP markers and those with a call rate below 90%. Whereas all birds had a call rate higher than 90% and passed the quality control criteria, the number of SNPs retained for analysis was 56 393 out of the 61 705 markers. beagle 5.0 (Browning *et al*. 2018) was used to phase genotypes and impute the missing genotypes.

### Statistical analyses

To trace the haplotype allele inheritance from parents to offspring in this turkey population, two parametrizations were considered in this study.

#### Allelic parametrization

Following Casellas *et al*. (2014, 2017), the probability of allele transmission (*p*) from heterozygous parents to offspring can be parameterized, including TRD effects on allelic basis, as:

$$p\left(A\right)=1‐p\left(B\right)=0.5+\alpha_{j}$$

$$p\left(B\right)=1‐p\left(A\right)=0.5‐\alpha_{j},$$

where A is the particular haplotype allele *j* being analysed, B represents the remaining haplotype alleles and *α_j_* is the overall TRD for the allele *j*. To capture parent‐specific TRD origin, a parent‐specific model was also implemented on the basis of allelic parametrization, but including two different parameters:

$$p_{s}\left(A\right)=1‐p_{s}\left(B\right)=0.5+\alpha_{sj}$$

$$p_{d}\left(A\right)=1‐p_{d}\left(B\right)=0.5+\alpha_{dj},$$

where s and d represent sire and dam respectively, and *α*_s_ *_j_* and *α*_d_ *_j_* are sire‐ and dam‐specific TRD for allele *j*. For all TRD parameters, flat priors were assumed within a parametric space ranging from −0.5 to 0.5. Under a Bayesian implementation, the conditional posterior probabilities of the TRD parameters are defined as:

$$p\left(\alpha_{j}|y\right)\propto p\left(y|\alpha_{j}\right)p\left(\alpha_{j}\right)$$

$$p\left(\alpha_{sj},\alpha_{dj}|y\right)\propto p\left(y|\alpha_{sj},\alpha_{dj}\right)p\left(\alpha_{sj}\right)p\left(\alpha_{dj}\right)$$

for overall and parent‐specific TRD respectively, where **y** is a column vector of genotypes of the offspring generation. The likelihood of data consists of a straightforward multiplication of the corresponding probabilities for each offspring (i.e. $\prod_{n}p_{\text{off}}\left(y_{i}\right)$), where *n* is the total number of offspring and *p*_off_ and *y_i_* are the probability and the genotype of the *i*th offspring respectively. The software trdscan version 1.0 (Id‐Lahoucine *et al*. 2019) uses a multinomial process, hence the likelihood of the data becomes:

$$\prod_{i=1}^{3}\frac{n_{i}!}{n_{\text{AA},i}!n_{\text{AB},i}!n_{\text{BB},i}!}\times p_{\text{off},i}{\left(\text{AA}\right)}^{n_{\text{AA},i}}\times p_{\text{off},i}{\left(\text{AB}\right)}^{n_{\text{AB},i}}\times p_{\text{off},i}{\left(\text{BB}\right)}^{n_{\text{BB},i}}.$$

In the above, *n_i_* is the sum of *n*_AA,_ *_i_*, *n*_AB,_ *_i_* and *n*_BB,_ *_i_* offspring genotypes, *p*_off,_ *_i_* is the probability of an offspring genotype from the *i*th mating and *n*_AA,_ *_i_*, *n*_AB,_ *_i_* and *n*_BB,_ *_i_* are the number of AA, AB and BB offspring genotypes from the specific *i*th mating respectively. For the parent‐specific TRD model, five kinds of matings were differentiated in the multinomial expression (i.e. $\prod_{i=1}^{5}$). For the sampling process, uniform proposal distributions (flat priors) were used for both *α*_g_ and *δ*_g_ within a deepened parametric space ranging from −1 to 1 (Casellas *et al*. 2012).

#### Genotypic parameterization

This parameterization captures the interaction between alleles of offspring genotypes. Additive (*α*_g_) and dominance (*δ*_g_) or over‐dominance, both positive or negative, TRD parameters are considered regardless of the origin of the allele. As described by Casellas *et al*. (2012, In press), the probability of observing offspring (*p*_off_) from heterozygous‐by‐heterozygous mating can be estimated as follows:

$$P_{\text{off}}\left(\text{AA}\right)=\frac{\left(1+\alpha_{gj}‐\delta_{gj}\right)}{4}$$

$$P_{\text{off}}\left(\text{AB}\right)=\frac{\left(1+\delta_{gj}\right)}{2}$$

$$P_{\text{off}}\left(\text{BB}\right)=\frac{\left(1‐\alpha_{gj}‐\delta_{gj}\right)}{4},$$

where *α*_g_ *_j_* and *δ*_g_ *_j_* are additive and dominance TRD parameters for the specific allele *j* respectively.

Under a Bayesian implementation, the conditional posterior probabilities of the TRD parameters are defined as:

$$p\left(\alpha_{gj},\;\delta_{gj}|y\right)\propto p\left(y|\alpha_{gj},\;\delta_{gj}\right)\;p\left(\alpha_{g}\right)\;p\left(\delta_{gj}|\alpha_{gj}\right),$$

where **y** is a column vector of genotypes of the offspring generation. For the sampling process, uniform proposal distributions (flat priors) were used for both *α*_g_ and *δ*_g_ within an extended parametric space ranging from −1 to 1 (Casellas *et al*. 2012). Thus, as the parametric space for *α*_g_ is initially [−1, 1], the parametric space for *δ*_g_ is restricted to [−1, |*α*_g_|]. Moreover, the parametric space of *α*_g_ itself is restricted to [−1 + *δ*_g_ to 1 − *δ*_g_] if *δ*_g_ > 0 and this guarantees that the sum of offspring’s genotypes probabilities for specific mating is equal to 1.

Transmission ratio distortion was evaluated using three sliding windows: 4, 10, and 20 SNP. The analyses were carried out using trdscan version 1.0 software (Id‐Lahoucine *et al*. 2019) based on a MCMC and the Metropolis–Hastings’ algorithm (Hastings 1970). A single MCMC of 100 000 iterations was run for each analysis with the first 10 000 iterations being discarded as burn‐in. Bayes factor (BF; Kass & Raftery 1995), which is a ratio of probabilities between full and null TRD models, was used to determine significant TRD (BF ≥ 100; decisive evidence according to Jeffreys’ (1984) scale). To obtain a reasonable statistical power and to minimize false TRD as a result of genotyping errors, only haplotype alleles with a minimum number of 50 informative offspring (i.e. from heterozygous parents) and five heterozygous sires and/or dams were analyzed.

The identified TRD regions were then filtered to minimize genotypic errors and to eliminate regions with random TRD. First, an approximate empirical null distribution of TRD (Id‐Lahoucine *et al*. 2019) at less than 0.001% margin error was used to remove TRD generated by chance. Also, a minimal number of informative parents (≥5 heterozygous sires and/or heterozygous dams) were considered to minimize possible false TRD from genotyping errors. Similarly, regions with few heterozygous parents fully explaining the observed TRD in the corresponding region were discarded as potential genotyping errors. Moreover, for the allelic parametrization, an arbitrary minimum magnitude of TRD at least 0.20 and the number of underrepresented offspring at least 1000 were considered to identify haplotype alleles with strong allelic TRD patterns. The number of underrepresented offspring is the total number of offspring expected, but not observed for a particular allele, which is also approximately equal to the number of informative offspring multiplied by twice the magnitude of the TRD. These thresholds ensured the identification of target haplotypes with a moderate‐to‐high level of TRD (i.e. moderate‐to‐high penetrance) as well as a reasonable number of offspring that are expected, but not observed (i.e. a minimum frequency for the allele in the population). For the genotypic parametrization, an arbitrary minimum number of 10 non‐observed homozygous offspring from heterozygous‐by‐heterozygous matings with additive TRD effect less than −0.50 and dominance TRD effect greater than 0.10 was considered to determine haplotype alleles with a substantial recessive TRD pattern. This threshold was considered to maintain the most important regions. It should also be emphasized that a well‐known lethal haplotype in cattle industry (Holstein haplotype 3) was initially identified by VanRaden *et al*. (2011) with only seven non‐observed homozygous offspring from heterozygous sires and heterozygous maternal grandsires matings. In addition, as different sliding windows were used, only the haplotype alleles with the largest BF within a region (with many physically linked haplotypes) were selected as the best candidates to explain the observed TRD in the region and potentially harbor the causal mutations. Thus, it is important to mention that the patterns of TRD observed in short windows are displayed in larger haplotypes including the same allele and also on other physically linked haplotypes, supporting the relevance of TRD in the corresponding particular locus.

### Functional and gene set enrichment

#### Assignment of lethal haplotypes to genes

Genes associated with complex traits are expected to represent only a small fraction of the genetic variation and, hence, some genetic variants with small effects and disease risks may not ever be detected (Peng *et al*. 2010; Abdalla *et al*. 2016). To further investigate the potential lethal haplotypes identified with TRD, the coordinates of these haplotype regions were used to mine for annotated genes using the Turkey 5.0 (release 102) assembly (Dalloul *et al*. 2010). It has been reported that strong LD may extend up to 10–30 kb in chickens (Rao *et al*. 2008; Megens *et al*. 2009; Qanbari *et al*. 2010). Thus, haplotypes were assigned to genes if they were located within the genomic sequence of an annotated gene or within 15 kb of the 5′ or 3′ ends of the first and last exons respectively. The 15 kb distance was used to capture proximal regulatory regions and other functional sites that may lie outside (e.g. promoter regions) but close to each gene. If a haplotype was found to be located within or close to more than one gene, all of these genes were included in the subsequent analyses.

#### Assignment of genes to functional categories

Gene Ontology (GO; Ashburner *et al*. 2000), Pathway Knowledgebase (Reactome; Fabregat *et al*. 2018) and Medical Subject Headings (MeSH; Coletti & Bleich 2001; Nelson *et al*. 2004) databases were used to define functional sets of genes. Biological descriptors, known as GO terms, fall into three categories: biological process, molecular functions and cellular components. Reactome, on the other hand, provides several biochemical networks including metabolic and regulatory pathways. Finally, MeSH is a collection of descriptors or headings representing key topics discussed in the papers indexed in the MEDLINE database. Whereas MeSH terms are classified into 19 categories, in this study, we were interested in only four: anatomy, disease, phenomena and processes, and lastly chemicals and drugs.

#### Pathway‐based association analysis

The Fisher’s exact test was used to declare the association of a given GO term, Reactome pathway and MeSH heading with TRD. This test was performed to search for an overrepresentation of significant genes in a given functional category among all genes. The *P*‐value of observing g significant genes in the term was calculated as follows:

$$P‐\text{value = 1}\;‐\sum_{i=0}^{g‐1}\frac{\left(\begin{array}{c} S\\ i \end{array}\right)\left(\begin{array}{c} N‐S\\ k‐i \end{array}\right)}{\left(\begin{array}{c} N\\ k \end{array}\right)}$$

where *N* is the total number of genes analyzed in the study, *S* is the total number of genes that were deemed significantly associated with TRD and *k* is the total number of genes in the functional category in the database under consideration.

Owing to a lack of biological information related to turkeys, both the turkey 5.0 assembly (Dalloul *et al*. 2010) and the GRCg6a chicken assembly (International Chicken Genome Sequencing Consortium 2004) were used to perform the pathway analysis. It is important to emphasize that all potential genes defined in this study are conserved across many organisms, including humans and chickens. The GO and Reactome enrichment analyses were carried out using GO (Ashburner *et al*. 2000) and panther (Mi *et al*. 2016) respectively, whereas the MeSH enrichment analysis was performed using the meshr package (Tsuyuzaki *et al*. 2015) available in the r environment (R Core Team 2019).

#### Gene network

The overlaps among significant genes associated with GO terms and Reactome pathways and their functional networks were also examined. We retrieved close neighbor genes and then generated an aggregate interaction network based on physical protein interaction and co‐expression of those genes using genemania software (Warde‐Farley *et al*. 2010).

## Results and discussion

### Prevalence of TRD across the turkey genome

**Figure 1:**
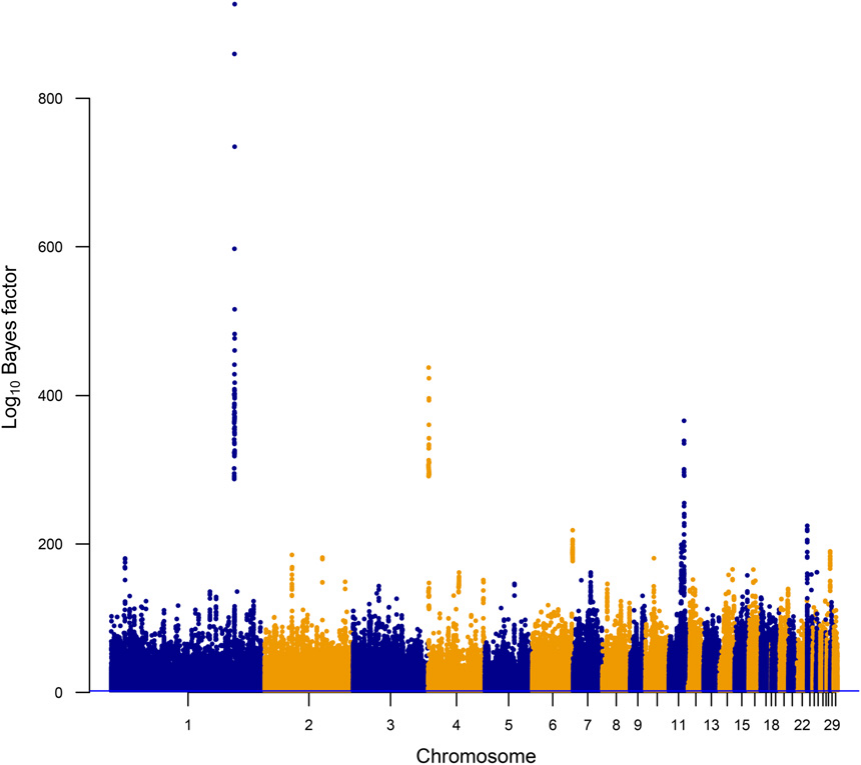
Bayes factor for haplotypes with transmission ratio distortion across the turkey genome. Significant haplotype alleles were determined based on log_10_ Bayes factor ≥2 according to Jeffreys’ scale (Jeffreys 1984)

The prevalence of TRD was widely distributed across the turkey genome as shown in Fig. 1. The initial numbers of haplotype alleles detected with decisive significant evidence (BF ≥ 100) of TRD, according to Jeffreys’ scale (Jeffreys 1984), were 48 951, 52 896 and 54 287 for 4, 10 and 20 SNP haplotype windows respectively. Despite this high number of regions, some of them had a large BF (10^100^) suggesting virtually 0 probability of the null TRD model. In addition, most of the detected TRD regions had low frequencies (i.e. rare variants); however, these haplotype alleles were supported by the large dataset. It is important to mention that it has been suggested that rare variants are more likely to be functional than common variants (Gorlov *et al*. 2008; Karaca *et al*. 2015), emphasizing the importance of TRD regions despite their low frequencies. It is noteworthy to mention that the majority of the regions were detected with more than one of the models applied, but with different fits and statistical significance. Thus, after the characterization of the TRD across the whole genome with the filtration criteria provided in the ‘Materials and methods’ section, the list of the most relevant haplotypes is provided in Tables 1 and 2 for regions with allelic and recessive TRD patterns respectively. These haplotypes indicate candidate regions potentially carrying deleterious alleles or genes affecting reproduction. However, these TRD findings were obtained under the assumption of no selection of offspring within a family to be genotyped. The violation of this must be taken into consideration by further investigating the source of the observed TRD signals. This is because the pre‐selection of offspring to be genotyped within families (Id‐Lahoucine & Casellas 2017) could be a source of bias on TRD analyses as discussed by Id‐Lahoucine *et al*. (2019). It is well known that selection of data has been a concern and a limitation for many types of analyses. In particular, for TRD analyses, major genes that present crucial and large impact in reproductive performance are targeted. The genetic selection performed in turkey populations is based on selection indexes targeting a multiple‐trait breeding objective (mainly highly polygenic production traits). Thus, the chance to observe TRD signals with absence of homozygous offspring as a result of selection is less likely. Here, we are using the TRD method as an alternative strategy to first scan possible relevant regions harboring lethal alleles that will require further research to exclude both genotyping errors and biases from pre‐selection in the offspring generation.

**Table 1:** Potential candidate lethal or semi‐lethal alleles identified with allelic transmission ratio distortion patterns by the allelic model

Chromosome	Region (kbp)	SNP^1^	Hetero sires^2^	Hetero dams^3^	Frequency (%)	AB^4^ × AA		AB × BB		AB × AB			TRD effects^6^		
						AA^5^	AB	AB	BB	AA	AB	BB	*α*	*α*_s_	*α*_d_
1	173 127–173 367	10	17	360	4.54	0	0	641	1688	1	5	15	−0.23	−0.33	−0.23
2	71 543–71 622	4	21	305	2.93	0	0	397	1664	0	7	2	−0.31	−0.21	−0.32
2	99 815–99 884	4	67	384	4.44	0	0	654	1915	0	27	39	−0.25	−0.25	−0.25
5	36 746–36 854	4	15	262	2.77	0	0	403	1505	0	2	3	−0.29	−0.31	−0.29
6	50 141–50 184	4	22	182	1.37	0	0	146	1242	0	1	11	−0.40	−0.44	−0.39
7	10 128–10 171	4	61	277	2.66	0	0	342	1344	0	14	42	−0.31	−0.26	−0.32
10	10 424–10 465	4	15	235	2.34	0	0	327	1338	0	5	6	−0.31	−0.38	−0.30
11	15 529–15 551	4	25	410	4.41	0	0	639	2157	0	10	22	−0.28	−0.16	−0.29
14	16 726–16 743	4	32	287	2.96	0	0	383	1557	0	9	9	−0.31	−0.31	−0.31
16	10 429–10 450	4	95	457	8.21	4	15	1136	2025	34	189	145	−0.15	−0.03	−0.22
23	69–81	4	27	92	1.31	0	0	189	1271	0	8	30	−0.38	−0.42	−0.30
28	4507–4549	10	29	264	2.8	0	0	372	1597	0	8	21	−0.32	−0.27	−0.32

^1^ Number of SNP in a haplotype window.

^2^ Number of heterozygous sires.

^3^ Number of heterozygous dams.

^4^ Genotypes of parents.

^5^ Genotypes of offspring.

^6^ *α*, *α*_s_ and *α*_d_ are overall, sire and dam allelic transmission ratio distortion respectively.

**Table 2:** Potential candidate lethal alleles identified with recessive transmission ratio distortion patterns by the genotypic model

Chr^1^	Region (Kbp)	SNP^2^	Hetero sires^3^	Hetero dams^4^	Frequency (%)	AB^5^ × AA		AB × BB		AB × AB			TRD effects^7^	
						AA^6^	AB	AB	BB	AA	AB	BB	*α*_g_	*δ*_g_
1	27 680–27 924	10	24	101	2.51	0	0	450	447	0	17	10	−0.58	0.29
1	29 253–29 484	10	30	108	4.33	0	0	305	265	0	20	11	−0.54	0.35
2	40 083–40 199	4	13	86	1.83	0	0	336	481	0	23	15	−0.77	0.14
3	29 955–30 188	10	5	39	0.67	0	0	106	142	0	15	10	−0.71	0.16
4	60 400–60 941	20	9	64	1.93	0	0	252	321	0	32	16	−0.72	0.21
4	65 823–65 908	4	10	48	0.91	0	0	153	232	0	20	11	−0.77	0.11
5	3900–3975	4	50	170	4.92	0	0	950	967	0	26	17	−0.62	0.30
6	8973–9097	10	19	81	2.48	0	0	431	458	0	21	11	−0.63	0.27
11	23 084–23 137	4	31	107	3.04	0	0	573	673	0	22	12	−0.67	0.23
12	15 689–15 829	20	9	49	1.84	0	0	285	304	0	15	10	−0.61	0.26
19	4054–4196	20	43	156	4.39	0	0	763	756	0	62	18	−0.63	0.33
21	9664–9680	4	38	129	3.03	0	0	525	573	0	52	22	−0.67	0.29
24	672–742	10	13	54	1.86	0	0	323	384	0	17	10	−0.66	0.22
28	1640–1794	20	20	107	2.96	0	0	484	476	0	15	10	−0.57	0.29

^1^ Chromosome.

^2^ Number of SNP on a haplotype window.

^3^ Number of heterozygous sires.

^4^ Number of heterozygous dams.

^5^ Genotypes of parents.

^6^ Genotypes of offspring.

^7^ *α*_g_ and *δ*_g_ are additive and dominance transmission ratio distortion respectively.

### Haplotypes with allelic TRD pattern using the allelic parametrization model

Across the turkey genome, 12 potentially lethal haplotype candidates were detected with allelic TRD, as shown in Table 1. The number of informative offspring detected in those 12 potentially lethal haplotype regions ranged between 1400 and 3548, whereas the number of underrepresented offspring reached 1564. Most of the haplotypes showed relatively small differences between male‐ and female‐specific TRD, supporting their unspecific‐parent TRD pattern. In contrast, one haplotype allele located on chromosome 16 showed parent‐specific TRD, where it was high for the dam (−0.22) and low via the sire (−0.03). Although this region had some offspring (4 and 15) from homozygous‐by‐heterozygous (AB × AA) matings (i.e. the homozygous parent carries two copies of the lethal allele), the numbers of offspring were extremely small compared with the expectations. Similarly, the matings of heterozygous‐by‐heterozygous (AB × AB) in this region produced some (34) homozygous (AA) individuals. However, this number is still substantially lower than expected, indicating lower viability. This could be due to the variation in specific TRD between males and females. As the probability of transmitting this lethal allele from heterozygous sires is close to the Mendelian expectation (0.5–0.03 = 0.47), the probability of observing live homozygous parents/offspring increases (4 instead of 0). Matings with either parent carrying the lethal allele in the homozygous state for the remaining 11 regions were not observed, supporting the lethal effect of this haplotype allele. On the other hand, it must be emphasized that these regions were also detected by the genotypic model. Nevertheless, the allelic model had better goodness‐of‐fit than the genotypic model in terms of deviance information criterion units and accurate TRD estimates with short credible intervals.

### Haplotypes with recessive TRD pattern using the genotypic parametrization model

Based on the genotypic parametrization model, 14 potentially lethal haplotypes were identified with additive‐ and dominance‐TRD resulting in lethal homozygous offspring from heterozygous‐by‐heterozygous matings as shown in Table 2. The additive TRD component of these regions ranged from −0.54 to −0.94, and interestingly, the negative effects of these haplotypes are counteracted by the dominance TRD effects, which ranged between 0.11 and 0.35. The interaction of additive and dominance TRD effects provides an equal chance for heterozygous (carriers) offspring to survive as non‐carrier birds. From the heterozygous‐by‐heterozygous (AB × AB) matings, no homozygous (AA) individuals were observed, indicating the lethality of the haplotypes in the homozygous state. For these regions, the numbers of carrier parents were between five and 50 for sires and between 39 and 170 for dams. The number of informative offspring ranged from 273 to 1960, whereas the number of expected homozygotes that were not observed ranged from 10 to 22. For a fully recessive TRD pattern, it is expected that there will be similar numbers of heterozygous (AB) and homozygous (BB) offspring from heterozygous‐by‐homozygous matings (AB × BB). Nevertheless, slightly more heterozygous offspring (AB) in heterozygous‐by‐homozygous matings were observed, which could be partly explained as a result of a random TRD (i.e. TRD generated by chance; Id‐Lahoucine *et al*. 2019).

It is worth noting that most of these regions were only detected with the genotypic model whereas no statistical evidence was found using the allelic model. This is due to their different parameterizations, where the genotypic model includes the interaction between alleles, allowing detection of recessive TRD patterns. Specifically, the negative effect of the additive TRD component can be contrasted with the positive effect of the dominance TRD component in heterozygous offspring, allowing for capture of recessive TRD patterns. In contrast, the fact that the allelic parametrization is based on targeting the transmission of an allele from a specific parent to offspring, which is separate from possible interaction in offspring generation, prevents its ability to capture recessive TRD patterns.

### Functional analysis and gene set enrichment

Sixty‐seven biological process, 17 cellular components, 35 molecular function GO terms, in addition to 19 Reactome pathways and nine MeSH terms showed a significant overrepresentation (*P*‐value < 0.05) of genes associated with TRD in turkeys. All of these significant terms and pathways are listed in Tables S1–S5. It is important to mention that these terms, pathways and genes should be further investigated and validated to avoid false positives. Four biological process GO terms, in a close GO hierarchy relationship and related to mitosis, showed a significant overrepresentation (*P*‐value < 0.05) of genes statistically associated with TRD (Table 3). *Mitotic spindle assembly checkpoint* (GO:0007094) is a *mitotic spindle checkpoint* (GO:0071174) and a *negative regulation of mitotic metaphase/anaphase transition* (GO:0045841), which is, in turn, a *negative regulation of mitotic sister chromatid separation* (GO:2000816). Mitosis, which is associated with TRD, is a specialized division of chromosomes that occur during the formation of reproductive cells (Nicklas 1971). A similar significant (*P*‐value < 0.05) cellular component GO term, *Mitotic spindle pole* (GO:0097431), was also associated with TRD (Table 4).

**Table 3:** Biological process function terms significantly overrepresented with genes statistically associated with transmission ratio distortion

GO ID	Term (GO hierarchy level)	Number of genes in the GO term	Number of significant genes	*P*‐value^1^
0051315	Attachment of mitotic spindle microtubules to kinetochore (10)	10	1	0.023
0033567	DNA replication, Okazaki fragment processing (11)	3	1	0.008
1902969	Mitotic DNA replication (11)	8	1	0.019
0007094	Mitotic spindle assembly checkpoint (14)	18	1	0.039
0071174	Mitotic spindle checkpoint (9)	18	1	0.039
2000697	Negative regulation of epithelial cell differentiation involved in kidney development (12)	2	1	0.006
2000094	Negative regulation of mesonephric nephron tubule epithelial cell differentiation (15)	1	1	0.004
0061218	Negative regulation of mesonephros development (11)	4	1	0.001
0045841	Negative regulation of mitotic metaphase/anaphase transition (12)	20	1	0.044
2000816	Negative regulation of mitotic sister chromatid separation (11)	21	1	0.046
0072183	Negative regulation of nephron tubule epithelial cell differentiation (14)	1	1	0.004
1903461	Okazaki fragment processing involved in mitotic DNA replication (12)	1	1	0.004
2000093	Regulation of mesonephric nephron tubule epithelial cell differentiation (12)	1	1	0.004

^1^ Significance declared at *P* < 0.05.

**Table 4:** Cellular component function terms significantly overrepresented in genes statistically associated with transmission ratio distortion

GO ID	Term (GO hierarchy level)	Number of genes in the GO term	Number of significant genes	*P*‐value^1^
0044444	Cytoplasmic part (7)	5503	18	0.038
0070176	DRM complex (15)	1	1	0.004
0034709	Methylosome (8)	6	1	0.014
0097431	Mitotic spindle pole (12)	18	1	0.039
0032021	NELF complex (14)	2	1	0.006
0090568	Nuclear transcriptional repressor complex (13)	19	2	0.000
0090571	RNA polymerase II transcription repressor complex (14)	4	2	0.000

^1^ Significance declared at *P* < 0.05.

Three GO terms detected in the analysis classified into the biological process domain showed significant association with TRD (Table 3). In addition to their similar functions in mitosis, these three terms are close in the GO hierarchy. *Okazaki fragment processing involved in mitotic DNA replication* (GO:1903461) is a *DNA replication, Okazaki fragment processing* (GO:0033567) and part of *mitotic DNA replication* (GO:1902969). In cell biology, Okazaki fragments (Sakabe & Okazaki 1966) comprise the processes involved in any mitotic cell cycle DNA replication, a necessary step in the cell cycle.

The *Negative regulation of mesonephric nephron tubule epithelial cell differentiation* (GO:2000094) term was detected as significantly enriched (*P*‐value < 0.01) with genes associated with TRD and related to cell differentiation. This GO term is close in the GO hierarchy and in function to four other GO terms, which all can be linked to TRD: GO:2000093, GO:0061218, GO:0072183 and GO:2000697. Cellular differentiation is the process in which a simple cell changes from one cell type to a more specialized type, which may occur in numerous steps (Jones & Taylor 1980; Slack 2007). In a study on the development of the kidney in mice, McCright *et al* (2001) reported that animals died perinatally owing to the lack of normal capillary tufts, which is a result of defects in kidney development.

The terms *NELF complex* (GO:0032021) and two similar GO terms, *methylosome* (GO:0034709) and *cytoplasmic part* (GO:0044444), are classified under the cellular component domain and show a significant overrepresentation of genes statistically associated with TRD (Table 4). The *NELF complex* is a key regulatory step of the transcription cycle (Tamborrini & Piatti 2019). The GO term *DRM complex* (GO:0070176), located in the cellular component category, is an *RNA polymerase II transcription repressor complex* (GO:0090571), which is, in turn, a *nuclear transcriptional repressor complex* (GO:0090568). Interestingly, a connection between RNA polymerase II and TRD has been previously reported (Paterson *et al*. 2009). Moreover, Harrison *et al*. (2006) reported that *DRM complex* is a transcriptional repressor complex and involved in cell fate specification.

Three GO terms, which are close in the GO hierarchy (Table 5), were classified into the molecular function category and showed a significant overrepresentation of genes statistically associated with TRD (Table 4). *Glycine:sodium symporter activity* (GO:0015375) is a *glycine transmembrane transporter activity* (GO:0015187), which is in turn a *neutral amino acid transmembrane transporter activity* (GO:0015175). Genetic factors that stop coding glycine cause late‐stage embryo lethality and hence TRD (Seidel *et al*. 2011). *Protein binding, bridging involved in substrate recognition for ubiquitination* (GO:1990756) is a *protein binding, bridging* (GO:0030674). The molecular function of binding and sperm motility was reported by Bauer *et al*. (2007). As the authors mentioned, the binding partner for Ropporin and Ras proteins to the outer surface of the dense fiber proteins plays a crucial function in sperm motility.

**Table 5:** Molecular function terms significantly overrepresented in genes statistically associated with transmission ratio distortion

GO ID	Term (GO hierarchy level)	Number of genes in the GO term	Number of significant genes	*P*‐value^1^
0005488	Binding (2)	9725	28	0.017
0015187	Glycine transmembrane transporter activity (13)	5	1	0.012
0015375	Glycine: sodium symporter activity (16)	1	1	0.004
0060090	Molecular adaptor activity (3)	124	2	0.028
0015175	Neutral amino acid transmembrane transporter activity (12)	23	1	0.048
0030674	Protein binding, bridging (4)	112	2	0.023
1990756	Protein binding, bridging involved in substrate recognition for Ubiquitination (11)	3	1	0.008

^1^ Significance declared at *P* < 0.05.

Two Reactome pathways were detected as significantly (*P*‐value < 0.01) enriched with genes related to TRD (Table 6). *Mismatch repair* (*MMR*)*directed by MSH2:MSH3* (*MutSbeta*; R‐GGA‐5358606) and *Mismatch Repair* (R‐GGA‐5358508) are similar in their activities to the GO term *Mitotic spindle assembly checkpoint* (GO:0007094) and represent the GO biological process *mismatch repair* (GO:0006298). Analogous to the GO terms related to mitosis and cell cycle, these two pathways are associated with DNA mismatch repair. It has been demonstrated that their activity increases and reaches the highest levels during the S phase of the cell cycle (Edelbrock *et al*. 2009). *Mismatch Repair* corrects single base mismatches and small insertion and deletion loops of unpaired bases. *Mismatch repair directed by MSH2:MSH3* (*MutSbeta*) binds unpaired loops of two or more nucleotides (Palombo *et al*. 1996).

**Table 6:** Reactome terms significantly overrepresented in genes statistically associated with transmission ratio distortion.

Reactome ID	Reactome term name	Number of genes in the pathway	Number of significant genes	*P*‐value^1^
R‐GGA‐5358508	Mismatch repair	11	1	0.025
R‐GGA‐5358606	Mismatch repair (MMR) directed by MSH2:MSH3 (MutSbeta)	3	1	0.008

^1^ Significance declared at *P* < 0.05.

A MeSH term, *Adenosine Monophosphate* (D000249), classified into the chemicals and drugs category, showed a significant overrepresentation of genes statistically associated with TRD (Table 7). Interestingly, various reproductive functions, such as those requiring hormone synthesis and maintenance of fluid composition, are modulated by adenosine (Zhou *et al*. 2006; Aliagas *et al*. 2010). Another significant (*P*‐value < 0.05) MeSH term, *Anaplasia* (D000708), is classified into the disease domain (Table 7). *Anaplasia* is related to neoplastic cells and refers to the loss of mature or specialized features of differentiated neoplastic cells (Rodriguez *et al*. 2010; Pujadas *et al*. 2019). Previous studies have shown that genetic variation among inbred mice as a result of continuous uploading and removal of rare variation (new mutations) may generate TRD (Casellas & Medrano 2008; Niu & Liang 2009).

**Table 7:** Disease and chemicals and drugs MeSH terms significantly overrepresented in genes statistically associated with transmission ratio distortion

Category	MeSH term ID	MeSH term name	*P*‐value^1^
Chemicals and drugs	D000249	Adenosine monophosphate	0.012
Disease	D000708	Anaplasia	0.048

^1^ Significance declared at *P* < 0.05.

### Gene network

**Figure 2:**
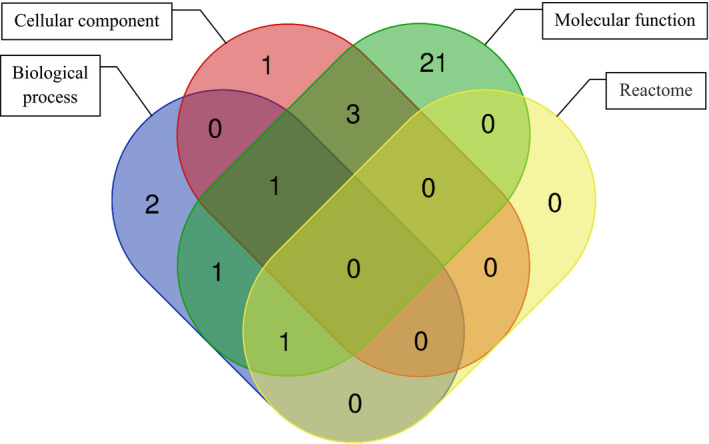
Venn diagram showing overlaps between significant genes associated with the terms of three GO domains (presented in Tables 3, 4, 5) and the Reactome pathways (presented in Table 6)

**Figure 3:**
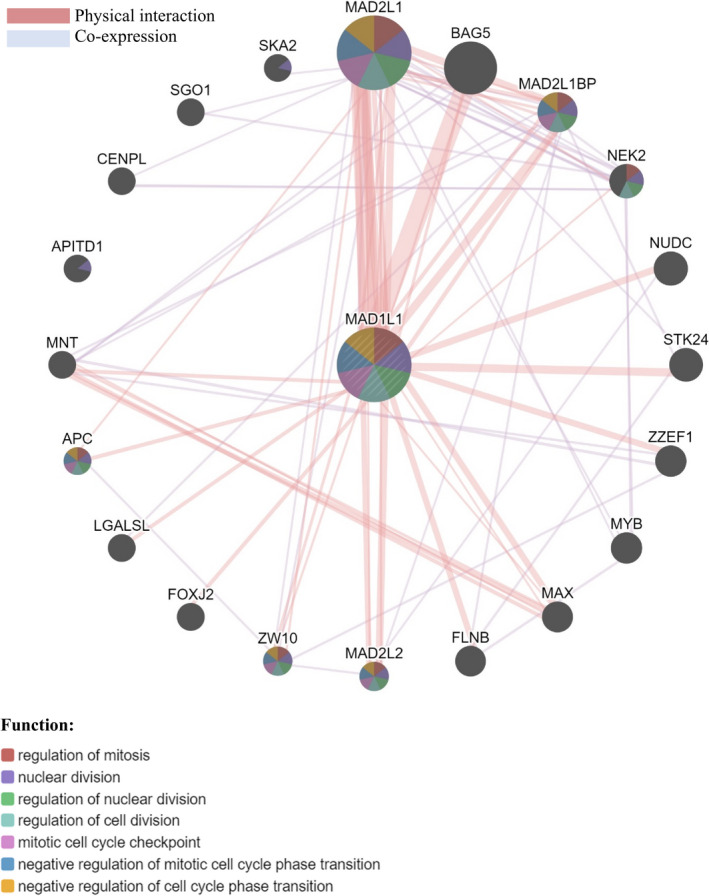
Network integration of gene MAD1L1 based on physical protein interaction and co‐expression. The gene APITD1 is associated with MAD1L1 through pathways (links are not shown)

**Figure 4:**
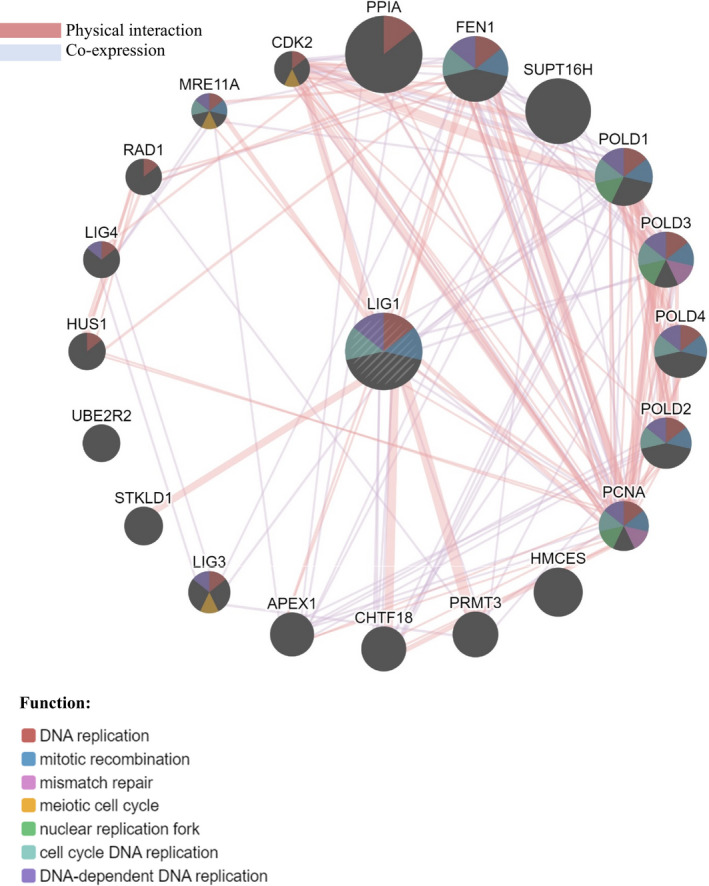
Network integration of gene LIG1 based on physical protein interaction and co‐expression. The gene UBE2R2 is associated with LIG1 through prediction (links are not shown)

The Venn diagram (Fig. 2) depicted the intersections between the significant genes associated with the terms of three GO domains (Tables 3, 4, 5) and the Reactome pathways (Table 6). Notably, the gene MAD1L1 is significant across the three GO domains and two of these GO domains (biological process and molecular function) overlap with the Reactome pathways in the gene LIG1. The aggregate interaction networks for MAD1L1 and LIG1 genes, based on physical protein interaction and co‐expression, are shown in Figs 3 and 4 respectively. The functional networks of gene MAD1L1 with 19 other genes indicate that this group of genes is involved in several activates related to mitosis such as regulation of mitosis and negative regulation of mitosis cell cycle phase transition. The gene APITD1 was not linked to MAD1L1 via physical protein interaction or co‐expression networks, but through the pathways network (this connection is not shown in Fig. 3). Similarly, the functional network for gene LIG1 revealed 19 genes associated with it through physical protein interaction and co‐expression networks (Fig. 4) and one gene (i.e. UBE2R2) through prediction. The functions for these genes include DNA replication and cell cycle DNA replication, which are among the most important cell activaties concerning reproduction and cell deviation.

## Conclusions

In this study, we applied allelic and genotypic parameterizations of TRD to detect potential lethal haplotypes in turkeys. The two methods revealed relevant regions across the turkey genome with either a classical recessive inheritance pattern (i.e. lethal only in the homozygous state) or allelic patterns (i.e. reduced viability of the carrier offspring). In addition to 19 Reactome pathways and nine MeSH terms, 67 biological process, 17 cellular components and 35 molecular function GO terms showed a significant (*P*‐value < 0.05) overrepresentation of genes statistically associated with TRD. Functional networks among several significant genes also showed links to mitosis and cell replication. These highlighted pathways and gene ontologies, along with the overall findings of this study, will contribute in developing novel turkey breeding and management strategies, as well as to specific mating programs for turkeys.

## Conflict of interests

All authors declare no conflict of interest.

## Funding

This study was conducted as part of the project entitled ‘Application of genomic selection in turkeys for health, welfare, efficiency and production traits’ funded by the Government of Canada through the Genome Canada Genomic Application Partnership Program and administered by Ontario Genomics (recipients: C.F. Baes (Academic), B.J. Wood (Industry)). This study was also financially supported by Hybrid Turkeys (Kitchener, Canada). The authors declare that this study received funding from Hybrid Turkeys. The funder was involved in providing the datasets used in this study.

## Supporting information


**Table S1** Biological process terms significantly overrepresented with genes statistically associated with TRD
**Table S2** Cellular component function terms significantly overrepresented with genes statistically associated with TRD
**Table S3** Molecular function terms significantly overrepresented with genes statistically associated with TRD
**Table S4** Reactome pathways significantly overrepresented with genes statistically associated with TRD
**Table S5** MeSH terms significantly overrepresented with genes statistically associated with TRD

## Data Availability

Data that support the findings of this study are available from Hybrid Turkeys upon reasonable request and were used under license for the current study, and thus are not publicly available.
