# Supplementary material for: Discovering lethal alleles across the turkey genome using a transmission ratio distortion approach
Source: Anim Genet. 2020 Oct 1;51(6):876–89. doi: 10.1111/age.13003 (PMC7702127; doi:10.1111/age.13003)
Supplement: Supplementary file 1 — Table S1 Biological process terms significantly overrepresented with genes statistically associated with TRD Table S2 Cellular component function terms significantly overrepresented with genes statistically associated with TRD Table S3 Molecular function terms significantly overrepresented with genes statistically associated with TRD Table S4 Reactome pathways significantly overrepresented with genes statistically associated with TRD Table S5 MeSH terms significantly overrepresented with genes statistically associated with TRD [file AGE-51-876-s001.docx]

Table S1. Biological process terms signiﬁcantly overrepresented with genes statistically associated with TRD.

| GO ID | Term | Number of genes in the GO term | Number of significant genes | P-value |
| --- | --- | --- | --- | --- |
| GO:0043651 | linoleic acid metabolic process | 1 | 1 | 0.004 |
| GO:1903804 | glycine import across plasma membrane | 1 | 1 | 0.004 |
| GO:0045636 | positive regulation of melanocyte differentiation | 3 | 1 | 0.008 |
| GO:0019219 | regulation of nucleobase-containing compound metabolic process | 2620 | 14 | 0.000 |
| GO:0080090 | regulation of primary metabolic process | 3882 | 16 | 0.005 |
| GO:0051171 | regulation of nitrogen compound metabolic process | 3797 | 16 | 0.004 |
| GO:0008380 | RNA splicing | 203 | 3 | 0.009 |
| GO:2000094 | negative regulation of mesonephric nephron tubule epithelial cell differentiation | 1 | 1 | 0.004 |
| GO:0072183 | negative regulation of nephron tubule epithelial cell differentiation | 1 | 1 | 0.004 |
| GO:2000697 | negative regulation of epithelial cell differentiation involved in kidney development | 2 | 1 | 0.006 |
| GO:2000093 | regulation of mesonephric nephron tubule epithelial cell differentiation | 1 | 1 | 0.004 |
| GO:1903461 | Okazaki fragment processing involved in mitotic DNA replication | 1 | 1 | 0.004 |
| GO:0033567 | DNA replication, Okazaki fragment processing | 3 | 1 | 0.008 |
| GO:0050678 | regulation of epithelial cell proliferation | 178 | 3 | 0.006 |
| GO:0001938 | positive regulation of endothelial cell proliferation | 49 | 2 | 0.005 |
| GO:2000696 | regulation of epithelial cell differentiation involved in kidney development | 13 | 1 | 0.029 |
| GO:0050794 | regulation of cellular process | 7442 | 23 | 0.035 |
| GO:0090185 | negative regulation of kidney development | 9 | 1 | 0.021 |
| GO:0048523 | negative regulation of cellular process | 2865 | 11 | 0.045 |
| GO:0072182 | regulation of nephron tubule epithelial cell differentiation | 9 | 1 | 0.021 |
| GO:0061218 | negative regulation of mesonephros development | 4 | 1 | 0.001 |
| GO:0061217 | regulation of mesonephros development | 18 | 1 | 0.039 |
| GO:0001504 | neurotransmitter uptake | 14 | 1 | 0.031 |
| GO:0015816 | glycine transport | 7 | 1 | 0.017 |
| GO:0089718 | amino acid import across plasma membrane | 9 | 1 | 0.021 |
| GO:0043090 | amino acid import | 11 | 1 | 0.025 |
| GO:1901360 | organic cyclic compound metabolic process | 2009 | 9 | 0.034 |
| GO:0006273 | lagging strand elongation | 5 | 1 | 0.012 |
| GO:0006271 | DNA strand elongation involved in DNA replication | 10 | 1 | 0.023 |
| GO:0022616 | DNA strand elongation | 14 | 1 | 0.031 |
| GO:1902969 | mitotic DNA replication | 8 | 1 | 0.019 |
| GO:0033260 | nuclear DNA replication | 19 | 1 | 0.042 |
| GO:0044786 | cell cycle DNA replication | 20 | 1 | 0.044 |
| GO:0045634 | regulation of melanocyte differentiation | 5 | 1 | 0.012 |
| GO:0060528 | secretory columnal luminar epithelial cell differentiation involved in prostate glandular acinus development | 4 | 1 | 0.01 |
| GO:0060525 | prostate glandular acinus development | 9 | 1 | 0.021 |
| GO:0060742 | epithelial cell differentiation involved in prostate gland development | 12 | 1 | 0.027 |
| GO:0042711 | maternal behavior | 5 | 1 | 0.012 |
| GO:0060746 | parental behavior | 6 | 1 | 0.014 |
| GO:0019098 | reproductive behavior | 16 | 1 | 0.035 |
| GO:0060770 | negative regulation of epithelial cell proliferation involved in prostate gland development | 5 | 1 | 0.012 |
| GO:0060768 | regulation of epithelial cell proliferation involved in prostate gland development | 9 | 1 | 0.021 |
| GO:0042127 | regulation of cell population proliferation | 865 | 5 | 0.037 |
| GO:0008285 | negative regulation of cell population proliferation | 338 | 3 | 0.036 |
| GO:0031284 | positive regulation of guanylate cyclase activity | 6 | 1 | 0.014 |
| GO:0030808 | regulation of nucleotide biosynthetic process | 16 | 1 | 0.035 |
| GO:0034244 | negative regulation of transcription elongation from RNA polymerase II promoter | 8 | 1 | 0.019 |
| GO:0034243 | regulation of transcription elongation from RNA polymerase II promoter | 22 | 1 | 0.048 |
| GO:0006357 | regulation of transcription by RNA polymerase II | 1481 | 8 | 0.012 |
| GO:0018242 | protein O-linked glycosylation via serine | 9 | 1 | 0.021 |
| GO:0002076 | osteoblast development | 13 | 1 | 0.029 |
| GO:0001503 | ossification | 145 | 2 | 0.039 |
| GO:0007094 | mitotic spindle assembly checkpoint | 18 | 1 | 0.039 |
| GO:0071174 | mitotic spindle checkpoint | 18 | 1 | 0.039 |
| GO:0031577 | spindle checkpoint | 18 | 1 | 0.039 |
| GO:0071173 | spindle assembly checkpoint | 18 | 1 | 0.039 |
| GO:0045841 | negative regulation of mitotic metaphase/anaphase transition | 20 | 1 | 0.044 |
| GO:1902100 | negative regulation of metaphase/anaphase transition of cell cycle | 22 | 1 | 0.048 |
| GO:2000816 | negative regulation of mitotic sister chromatid separation | 21 | 1 | 0.046 |
| GO:0043484 | regulation of RNA splicing | 110 | 2 | 0.023 |
| GO:0050679 | positive regulation of epithelial cell proliferation | 94 | 2 | 0.017 |
| GO:0072074 | kidney mesenchyme development | 10 | 1 | 0.023 |
| GO:0051315 | attachment of mitotic spindle microtubules to kinetochore | 10 | 1 | 0.023 |
| GO:0051103 | DNA ligation involved in DNA repair | 7 | 1 | 0.017 |
| GO:0006266 | DNA ligation | 10 | 1 | 0.023 |
| GO:0001936 | regulation of endothelial cell proliferation | 72 | 2 | 0.01 |
| GO:0060612 | adipose tissue development | 18 | 1 | 0.039 |

Table S2. Cellular component function terms signiﬁcantly overrepresented with genes statistically associated with TRD.

| GO ID | Term | Number of genes in the GO term | Number of significant genes | P-value |
| --- | --- | --- | --- | --- |
| GO:0070176 | DRM complex | 1 | 1 | 0.004 |
| GO:0090571 | RNA polymerase II transcription repressor complex | 4 | 2 | 0.000 |
| GO:0090568 | nuclear transcriptional repressor complex | 19 | 2 | 0.000 |
| GO:0017053 | transcriptional repressor complex | 51 | 2 | 0.005 |
| GO:0044424 | intracellular part | 10298 | 29 | 0.040 |
| GO:0005622 | intracellular | 10298 | 29 | 0.040 |
| GO:0043226 | organelle | 9059 | 27 | 0.019 |
| GO:0043229 | intracellular organelle | 8837 | 26 | 0.028 |
| GO:0043231 | intracellular membrane-bounded organelle | 7338 | 23 | 0.023 |
| GO:0043227 | membrane-bounded organelle | 7787 | 24 | 0.025 |
| GO:0032021 | NELF complex | 2 | 1 | 0.006 |
| GO:0034709 | methylosome | 6 | 1 | 0.014 |
| GO:0044444 | cytoplasmic part | 5503 | 18 | 0.038 |
| GO:0044615 | nuclear pore nuclear basket | 7 | 1 | 0.017 |
| GO:0097431 | mitotic spindle pole | 18 | 1 | 0.039 |
| GO:0005753 | mitochondrial proton-transporting ATP synthase complex | 17 | 1 | 0.037 |
| GO:0005794 | Golgi apparatus | 924 | 5 | 0.046 |

Table S3. Molecular function terms signiﬁcantly overrepresented with genes statistically associated with TRD.

| GO ID | Term | Number of genes in the GO term | Number of significant genes | P-value |
| --- | --- | --- | --- | --- |
| GO:0004074 | biliverdin reductase activity | 1 | 1 | 0.004 |
| GO:0016628 | oxidoreductase activity, acting on the CH-CH group of donors, NAD or NADP as acceptor | 14 | 1 | 0.030 |
| GO:0015375 | glycine:sodium symporter activity | 1 | 1 | 0.004 |
| GO:0015187 | glycine transmembrane transporter activity | 5 | 1 | 0.012 |
| GO:0015175 | neutral amino acid transmembrane transporter activity | 23 | 1 | 0.048 |
| GO:0005295 | neutral amino acid:sodium symporter activity | 1 | 1 | 0.004 |
| GO:0005283 | amino acid:sodium symporter activity | 11 | 1 | 0.024 |
| GO:0005343 | organic acid:sodium symporter activity | 21 | 1 | 0.045 |
| GO:0005416 | amino acid:cation symporter activity | 14 | 1 | 0.030 |
| GO:0008048 | calcium sensitive guanylate cyclase activator activity | 2 | 1 | 0.006 |
| GO:0030250 | guanylate cyclase activator activity | 3 | 1 | 0.008 |
| GO:0030249 | guanylate cyclase regulator activity | 4 | 1 | 0.010 |
| GO:0010851 | cyclase regulator activity | 4 | 1 | 0.010 |
| GO:0010853 | cyclase activator activity | 3 | 1 | 0.008 |
| GO:1990756 | protein binding, bridging involved in substrate recognition for ubiquitination | 3 | 1 | 0.008 |
| GO:0030674 | protein binding, bridging | 112 | 2 | 0.023 |
| GO:0060090 | molecular adaptor activity | 124 | 2 | 0.028 |
| GO:0005488 | binding | 9725 | 28 | 0.017 |
| GO:0003910 | DNA ligase (ATP) activity | 4 | 1 | 0.010 |
| GO:0003909 | DNA ligase activity | 5 | 1 | 0.012 |
| GO:0016886 | ligase activity, forming phosphoric ester bonds | 9 | 1 | 0.020 |
| GO:0008327 | methyl-CpG binding | 10 | 1 | 0.022 |
| GO:1901363 | heterocyclic compound binding | 4595 | 16 | 0.025 |
| GO:0097159 | organic cyclic compound binding | 4654 | 16 | 0.026 |
| GO:0003677 | DNA binding | 1659 | 9 | 0.006 |
| GO:0003676 | nucleic acid binding | 2786 | 13 | 0.005 |
| GO:0001228 | DNA-binding transcription activator activity, RNA polymerase II-specific | 272 | 3 | 0.019 |
| GO:0032561 | guanyl ribonucleotide binding | 361 | 3 | 0.040 |
| GO:0019001 | guanyl nucleotide binding | 361 | 3 | 0.040 |
| GO:0001883 | purine nucleoside binding | 346 | 3 | 0.036 |
| GO:0001067 | regulatory region nucleic acid binding | 783 | 5 | 0.023 |
| GO:0044212 | transcription regulatory region DNA binding | 782 | 5 | 0.023 |
| GO:0000977 | RNA polymerase II regulatory region sequence-specific DNA binding | 650 | 4 | 0.046 |
| GO:0001012 | RNA polymerase II regulatory region DNA binding | 656 | 4 | 0.047 |
| GO:0003682 | chromatin binding | 390 | 3 | 0.048 |

Table S4. Reactome pathways significantly overrepresented with genes statistically associated with TRD.

| Reactome ID | Reactome term name | Number of genes in the pathway | Number of significant genes | P-value |
| --- | --- | --- | --- | --- |
| R-GGA-418038 | Nucleotide-like (purinergic) receptors | 16 | 1 | 0.035 |
| R-GGA-417973 | Adenosine P1 receptors | 4 | 1 | 0.010 |
| R-GGA-1169092 | Activation of RAS in B cells | 6 | 1 | 0.014 |
| R-GGA-189445 | Metabolism of porphyrins | 14 | 1 | 0.031 |
| R-GGA-5358508 | Mismatch Repair | 11 | 1 | 0.025 |
| R-GGA-5358606 | Mismatch repair (MMR) directed by MSH2:MSH3 (MutSbeta) | 3 | 1 | 0.008 |
| R-GGA-110362 | POLB-Dependent Long Patch Base Excision Repair | 6 | 1 | 0.014 |
| R-GGA-110373 | Resolution of AP sites via the multiple-nucleotide patch replacement pathway | 20 | 1 | 0.044 |
| R-GGA-69190 | DNA strand elongation | 22 | 1 | 0.048 |
| R-GGA-2514859 | Inactivation, recovery and regulation of the phototransduction cascade | 17 | 2 | 0.000 |
| R-GGA-69186 | Lagging Strand Synthesis | 18 | 1 | 0.039 |
| R-GGA-5674135 | MAP2K and MAPK activation | 38 | 2 | 0.003 |
| R-GGA-5358565 | Mismatch repair (MMR) directed by MSH2:MSH6 (MutSalpha) | 10 | 1 | 0.023 |
| R-GGA-8956321 | Nucleotide salvage | 20 | 1 | 0.044 |
| R-GGA-69183 | Processive synthesis on the lagging strand | 13 | 1 | 0.029 |
| R-GGA-8849471 | PTK6 Regulates RHO GTPases, RAS GTPase and MAP kinases | 16 | 1 | 0.035 |
| R-GGA-180336 | SHC1 events in EGFR signaling | 8 | 1 | 0.019 |
| R-GGA-187687 | Signalling to ERKs | 24 | 2 | 0.001 |
| R-GGA-2514856 | The phototransduction cascade | 19 | 2 | 0.000 |

Table S5. MeSH terms significantly overrepresented with genes statistically associated with TRD.

| Category | MeSH term ID | MeSH term name | P-value |
| --- | --- | --- | --- |
| Anatomy | D001402 | B-Lymphocytes | 0.042 |
| Chemicals and Drugs | D000249 | Adenosine Monophosphate | 0.012 |
| Phenomena and Processes | D000221 | Adaptation, Ocular | 0.002 |
|  | D000782 | Aneuploidy | 0.012 |
| Disease | D000267 | Tissue Adhesions | 0.006 |
|  | D000848 | Anodontia | 0.010 |
|  | D000015 | Abnormalities, Multiple | 0.018 |
|  | D000013 | Congenital Abnormalities | 0.032 |
|  | D000708 | Anaplasia | 0.048 |
